# Utilisation of evidence from Thailand’s National Health Examination Survey in policy development: finding the weakest link

**DOI:** 10.1186/s12961-019-0512-4

**Published:** 2019-12-26

**Authors:** Sripen Tantivess, Jomkwan Yothasamut, Wilailak Saengsri

**Affiliations:** Department of Health, Ministry of Public Health, Health Intervention and Technology Assessment Program, 6th Floor, 6th Building, Nonthaburi, 11000 Thailand

**Keywords:** Thailand, National Health Examination Survey, evidence utilisation, policy development

## Abstract

**Background:**

Health surveillance and survey data are helpful in evidence-informed policy decisions. This study is part of an evaluation of the National Health Examination Survey (NHES) programme in Thailand. This paper focuses on the obstacles in the translation of survey information into policies at a national level.

**Methods:**

In-depth interviews with relevant individuals and representatives of institutes were carried out for the data collection. A total of 26 focal informants included executives and staff of NHES funders, government health agencies, civil society organisations, health experts, NHES programme managers and researchers in the survey network.

**Results:**

Utilisation of NHES data in policy-making is limited for many reasons. Despite the potential users’ positive views on the technical integrity of experts and practitioners involved in the NHES, the strength of employing health examinations in the data collection is not well recognised. Meanwhile, alternative health surveillance platforms that offer similar information on a shorter timescale are preferable in policy monitoring and evaluation. In sum, the lack of governance of Thailand’s health surveillance system is identified as a key element hindering the translation of health surveys, including the NHES, into policies.

**Conclusion:**

Despite an adequate capacity to conduct population health surveys, the lack of governance structure and function has resulted in a fragmented health monitoring system. Large and small survey projects are conducted and funded by different institutes without common policy direction and alignment mechanisms for prioritising survey topics, collective planning and capacity-building programmes for survey practitioners and users. Lessons drawn from Thailand’s NHES can be helpful for policy-makers in other low- and middle-income countries, as effective governance for evidence generation and utilisation is necessary in all contexts, regardless of income level and available resources.

## Key messages

A case study of the utilisation of Thailand’s National Health Examination Survey (NHES) emphasised the importance of evidence utilisation to support decision-making in health policy and resource allocation in resource-limited settings. Utilisation of NHES data in policy-making is limited. The lack of governance of Thailand’s health surveillance system is identified as a key element hindering the translation of health surveys, including the NHES, into policies.

## Introduction

Population health surveys generate helpful information that contributes to the monitoring of health risk behaviours and disease burden. The main objective of these surveys is to offer high quality data for resource allocation, planning and programme evaluation [1]. Despite such benefits, the availability and utilisation of such information are limited in low- and middle-income countries (LMICs) [2, 3]. Resource constraints and the lack of technical and management capacity have been identified as major obstacles in conducting regular national health surveys that capture policy-relevant topics. In some settings, survey data exist, but their role in driving policy decisions is absent due to several reasons [4, 5]. The literature focusing on the use of scientific evidence, including survey data, in health policy is relatively rich, describing the roles of responsible institutes, funding agencies, policy authorities and stakeholders [6–8]. This paper reviews an experience in Thailand, where the use of results from its population health monitoring project in policy-making has been constrained by inadequate management and a range of contextual factors.

### Health examination surveys: advantages and contributions to policy

Health examination surveys have been introduced in many high-income countries, for example, Australia, England, Finland, Germany, Japan, Korea and the United States. This form of health monitoring is also conducted in LMICs, including Brazil, Malaysia, South Africa and Thailand. The major strengths of these programmes include nationwide samples as well as physical and laboratory examinations employed during data collection. Owing to these features, the surveys yield relatively comprehensive, reliable information on population health status, disease prevalence, and the distribution of health problems and risk behaviours when compared with other sources, such as self-reports of illnesses in interview surveys, reviews of medical records and routine reports by health providers [9, 10]. It is evident that health examination surveys substantially contribute to policy development and programme design, as shown in the case of the Health Survey for England (HSE) and the United States National Health and Nutrition Examination Survey (NHANES). Data from the HSE have been used in the stages of problem identification, policy formulation and programme review concerning the control of obesity, chronic kidney disease, hypertension, diabetes mellitus and iron deficiency anaemia [11, 12]. In the United States, NHANES data were translated into policy to eliminate lead from gasoline as well as food and soft drink cans, and the data were instrumentally used in the development of evaluative growth charts for children employed by health workers [13]. In addition, the NHANES raised awareness of public health institutes and concerned parties on the rising trend of non-communicable diseases (NCDs), which prompted attention to appropriate interventions [14]. In LMICs, although academic literature and government documents emphasise the potential use of national survey data to inform health priority-setting and planning, evidence on their contribution to particular policy decisions is limited [15–18].

### Thailand’s National Health Examination Survey and the 2016 evaluation

In Thailand, the National Health Examination Survey (NHES) has been conducted periodically – every 5 years – since 1991 [19, 20]. The Policy and Planning Division from the Ministry of Public Health (MOPH) played a leading role in NHES I and NHES II. From the third cycle onward, a specialised office affiliated with the Health Systems Research Institute (HSRI) – an autonomous government unit for health research coordination and knowledge management – has been placed in charge of the survey. A network of university lecturers in the country’s four regions and Bangkok (the capital) are responsible for data collection, coordination with hospital laboratories and quality assurance at the survey sites. Sixty million Thai baht (USD 1.7 million) was jointly granted for the fifth cycle of the NHES by three agencies, namely the MOPH, HSRI and the Thai Health Promotion Foundation (ThaiHealth). From 2012 to 2016 the survey under the NHES V project involved almost 19,500 participants – from 1 to over 60 years of age – in physical examinations, laboratory analyses and interviews. A wide range of topics on socioeconomic status, health problems and risk behaviours were covered in this survey.

Commissioned by ThaiHealth in 2016, an evaluation of the NHES was conducted by the Health Intervention and Technology Assessment Program (HITAP) – an independent research unit in the MOPH. The findings indicated that a crucial weakness of health surveys in Thailand included the lack of a governance structure and policy to integrate all existing survey projects that were introduced and funded by different institutes, resulting in the fragmented governing system of survey programmes [21]. As such, no long-term plan was established for institutional coordination, human resources development and survey quality improvement. Many survey topics on chronic NCDs and risk behaviours, such as alcohol consumption, tobacco consumption and physical inactivity, were duplicated. This evaluation revealed that NHES’ performance has been affected not only by such contextual elements but also by policy and management measures within the survey office.

Built on the aforementioned NHES evaluation study, this paper discusses the translation of the survey data into policies by exploring the NHES’s policy and key determinants of the connection between the survey and policy-making sphere in view of NHES funding agencies, potential users, and survey programme managers and practitioners.

### Conceptual framework

To gain insight into the use of NHES data to inform policy, a conceptual framework was devised to capture three groups of elements as suggested by Court and Young, namely the nature of evidence, political context, and links between the research community and policy-making institutes [8]. We transferred the concepts from this framework to our assumption that the utilisation of NHES data in policy development is influenced by the users’ perceptions of the quality of the survey, which involves not only the reliability of the methodology and approaches, but also the credibility of the survey practitioners and how the survey results are communicated. The second set of potential factors comprises policy processes with a focus on the prevailing culture and experiences of evidence-informed decision-making in Thailand’s health system. A third set of factors involved the relationship between the users of NHES data and the office in charge of the survey. We also integrated the concepts of research uptake and research use proposed by Morton et al. as components of their Research Impact Assessment framework [22] to understand the situation and explore factors affecting the utilisation of the survey’s outputs in the policy area. These include resources and effort as inputs to link the NHES with policy, policy-makers’ awareness of the utility of data, and their capacity to use such information and related evidence.

## Methods

### Data collection

The data collection for NHES programme evaluation was conducted between November 2016 and April 2017. In order to gather the information on a breadth of roles and experiences related to the conduct of NHES and use of the survey data, key informants were purposively selected, including executives and administrative personnel in NHES funding agencies, national health policy authorities, survey practitioners, respective health experts and researchers involved in this population health survey. We also employed a snowball sampling technique for this evaluation recruiting additional participants recommended by other informants.

A small-scale questionnaire survey was carried out with three executives of the NHES funding agencies and the NHES’ principal investigator to explore their perspectives and expectations concerning this health examination survey such as its return on investment and future development. The information obtained was then analysed and used to guide the formulation of in-depth interview questions. The interviews aimed to explore (1) key stakeholders’ perspectives on the quality of the NHES and their experiences on evidence-informed policy; (2) collaborations between NHES project managers, survey practitioners and data users in different policy areas; and (3) resources and efforts as inputs to link the NHES with policy, policy-makers’ awareness on the utility of the survey data and their capacity to use such evidence.

In-depth interviews were conducted with 26 informants. These included NHES project coordinators (*n* = 2), researchers in the survey network (*n* = 4), health officials in the MOPH (*n* = 7), health experts in particular areas from the academic sector, civil society organisations and royal colleges addressed in the survey (*n* = 7), and managers of NHES funding agencies (*n* = 6). All interview sessions were conducted in Thai, and digitally audio recorded with informed consent from respondents. Verbatim transcription was performed by a group of independent, experienced transcribers.

#### Data analysis approach and quality assurance

In this study, information from in-depth interviews and document review was analysed using a content analysis approach in accordance with the four themes set in the conceptual framework. Triangulation was employed to determine the concordance and variations as well as to examine validity through the convergence of information from distinct sources [23]. Furthermore, a stakeholder consultation session was held on March 3, 2017, at HITAP office with the purpose of reviewing the preliminary results and discussing policy recommendations [24]. Key stakeholders that participated in the meeting included representatives from the NHES project and funding agencies, and potential users of the survey information.

#### Research ethics

This study was approved by the Ethics Committee of the Institute for the Development of Human Research Protections (IHRP), Thailand, on October 3, 2016 (IHRP.1079/2559). The anonymity and confidentiality of informants’ identities are guaranteed.

## Results

### Use of NHES data in policy development

Executives and staff of NHES funding agencies and technical officers in MOPH departments shared the common perspective that, since this series of health surveys have been conducted by credible principal investigators and research faculties, they provide valuable information for policy decisions. A division director at ThaiHealth and a senior health officer argued that NHES is the country’s largest regular health survey. More importantly, it is the only population-based survey that employs physical and laboratory examinations together with structured interviews for data collection. As pointed out by the senior officer, the MOPH introduced a countrywide diabetes screening programme because data from the NHES IV in 2008–2009 indicated that a significant portion of Thai people living with diabetes did not seek proper care; hence, this health problem had not been detected. The officer also emphasised that, without the laboratory analyses of blood glucose, hidden cases of diabetes would have never been uncovered. This was the only anecdote illustrating the use of NHES data in policy-making.

It is noteworthy that other interviewees did not provide concrete illustrations of the role of the NHES in the agenda-setting stage and subsequent policy changes. The Director of a Civil Society Organisation (CSO) asserts that their mission to strengthen healthy lifestyles and advocate for related public policies is usually informed by evidence, including epidemiological data. The Director further argues, however, that the NHES is not the only data source that captures topics of the organisation’s interest; in some instances, data from other surveys are more relevant than the NHES in policy advocacy. For example, for cigarette smoking, there are several surveys conducted by the National Statistical Office and Department of Disease Control that offer similar information, such as the Health and Welfare Survey, Cigarette Smoking and Alcohol Drinking Behavior Survey, and the Behavior Risk Factor Surveillance System. The Director is of the opinion that, with regards to the health topics that they work on, all of these surveys, including the NHES, are indistinct since they employ structured interviews as the method for data collection. In other words, in such a case where the utilised data were not derived from physical and laboratory examinations, the NHES does not pose any comparative advantage over other surveys. The Director’s organisation occasionally demands further analysis of NHES data, e.g. determining the prevalence of health problems and risk behaviours in specific age groups and geographical areas, because the results are helpful for strategic development and planning. However, as this interviewee further points out, most of the requests for additional analysis of data are not addressed because such technical exercises are carried out based on the interests of researchers.

This study indicates that NHES’ contribution to the monitoring and evaluation (M&E) of national policy is limited. Currently, performance assessments of MOPH programmes require information from routine facility-based datasets on service delivery [25]. This is in line with the recommendations from a study commissioned by the HSRI in 2013. Aiming to address the Health Secretary’s concern on the reliability of data for health promotion and disease prevention policy, that study reviewed output and outcome indicators in 10 areas used by the MOPH and National Health Security Office – the health insurance manager for three-quarters of the Thai population [26]. It was revealed that NHES data match with certain indicators in four areas, including the screening of chronic diseases and risk behaviours, cancer screening, nutrition surveillance and age-related health screening. The review also argued that the MOPH’s facility-based reporting system is the most appropriate data platform for M&E, and the results of population-based surveys can be used for verification purposes. Despite these findings, the NHES is more favourable than other countrywide surveys in two out of the four areas because of its rigor study design.

Financed by earmarked excise alcohol and tobacco taxes, ThaiHealth is a major funding institute for the development of health promotion programmes and innovations, including the NHES. According to its long-term strategy for 2012 to 2021, achievements in 10 critical areas are being pursued as strategic targets [27]. These areas include cigarette smoking, alcohol consumption, HIV infection in pregnant women, fruits and vegetable consumption, physical activity, child overweight and obesity, mortality associated with road traffic accidents, the population’s happiness, affectionateness of the family, and a strong community. Although the NHES provides information relevant to three indicators, namely the prevalence of cigarette smoking, alcohol drinking and physical activity, ThaiHealth uses corresponding data from the Cigarette Smoking and Alcohol Drinking Behavior Survey and the Physical Activity Survey instead. Therefore, data from the NHES have contributed to the M&E of ThaiHealth’s mission in only two areas – fruit and vegetable consumption, and child overweight and obesity.

### Why are NHES data rarely used in policy monitoring and evaluation?

Interviews with key informants suggest that the main reason for not using NHES data among policy-makers and technical officers is the existence of other surveys that have similar health topics as well as the perception that the quality of these alternatives is almost the same. Meanwhile, a few of the informants argued that they recognise the differences between the NHES and other surveys in terms of sample, methodology, quality control, project management, reliability and usefulness. Although they trust the quality of the NHES, findings from other surveys are employed since NHES data are not available in a timely manner as the survey is carried out on a 5-year basis. In the case of physical activity policy, for example, the office in the MOPH responsible for said policy has collaborated with a university’s research institute to conduct a series of surveys in this area since 2012, even though physical activity is a topic captured in the NHES and the Disease Control Department’s Behavior Risk Factor Surveillance System [28]; as argued by a respective health officer, this is because the latter two surveys cannot meet the demand for an annual evaluation of the respective programmes.

As mentioned previously, a study to identify appropriate data sources for the M&E of health promotion and disease prevention policies recommended the use of data from the MOPH as a routine reporting system [26]. The review analysed the strengths and weaknesses of potential data platforms and suggested that, although the current facility-based dataset has substantial deficits, this system is much more comprehensive than any other source of information, especially in terms of the continuity of data and coverage of policy-relevant parameters. Regarding nationwide health surveys, including the NHES, the review indicated that their strengths are in the methodology and quality assurance, and some of the surveys include demographic and socioeconomic factors that are helpful for further analysis. However, these surveys possess crucial drawbacks as they are time-consuming, and the results cannot be released in line with the annual administration policy cycle. As such, it was recommended that the MOPH should strengthen its routine reporting system in certain aspects, including establishing a mechanism for verification of the reported data against reliable survey findings.

### Resource and management constraints and lack of health survey system governance

NHES funders, the principal investigator, project managers and data users perceived that the lack of resources and inadequate management hindered the translation of NHES data into policies. At the same time, these informants pointed out that their institutes should not be responsible for advocating the use of NHES findings in policy and practice. As argued by a programme director in ThaiHealth, affiliated units of his institute often utilise NHES data and, thus, the organisation provides financial support to the survey. However, a very limited amount of the budget can be allocated to NHES policy integration activities as they are beyond ThaiHealth’s mandate. With regard to the MOPH, although it is the national health authority and a co-funder of the NHES, its role in facilitating data utilisation, and even the M&E of the survey’s progress and performance, is limited due to frequent changes in the director of the office. One of the survey practitioners at the regional level asserted that when he joined the fourth and fifth rounds of the NHES, the MOPH along with its provincial offices and health facilities did not play an active role in facilitating data collection and analysis, and he could only obtain collaboration from health officials and providers in certain areas owing to personal relationships. Meanwhile, changes in HSRI leadership and policy were claimed as the cause of human resources shortages and inadequate management in the NHES office during the latest cycle of this survey from 2012 to 2015.

From a broader perspective, the need to institutionalise the NHES and the lack of governance of the country’s health survey system have been discussed in NHES reports since the early phase of this survey programme [29, 30]. Previous efforts to establish an institutional structure, functions and strategy for a sustainable NHES occurred in 2001, when the survey programme was transferred from the MOPH to an HSRI-affiliated office [30]. However, owing to policy shifts in the HSRI in 2016, there was a plan to transfer this survey programme again to a research unit in a medical school in Bangkok. In addition, since the first round of NHES conducted in 1991, there has been no medium- or long-term plan for this survey and its budget is not stable, as the head programme manager has to seek grants from different agencies for the survey in each cycle. Some informants pointed out that the absence of a country-level governing mechanism to coordinate key actors for survey policy development has resulted in crucial deficits. These include, for instance, unnecessary duplication of some survey topics and spending on survey tools and equipment that can be shared across projects, and the use of non-standardised approaches or measurements.

### What are the weakest links of the NHES-policy connection?

To identify the weakest and most crucial links for the use of the NHES to inform policy, this section draws on the findings discussed previously. The first deficit is based on our observation that the NHES’ notable strength, i.e. the use of physical and laboratory examinations, was seldom mentioned when the interviewees talked about this survey. In addition, a Civil Society Organization (CSO) executive was the only respondent who recognised this feature of survey data as beneficial to their mission and raised concerns on the unmet demand for policy-relevant NHES outputs, i.e. further analysis of the survey data. This suggests that some decision-makers and technical staff may have limited knowledge and understanding of the features of the NHES data and other population-based survey programmes. These problems may also result from the resource constraints for NHES dissemination campaigns as well as the lack of a proper mechanism to identify and respond to policy questions that can be addressed by additional analysis of the NHES data. Furthermore, the fact that the NHES could not deliver its results to policy-makers in a timely manner is another factor that hinders the use of the survey data in the policy-making process. As such, it could be argued that a critical shortfall involves the link between the policy-making community and institutes responsible for the survey in various interrelated aspects because inadequate resources and management may have adverse effects not only on NHES dissemination but also on the performance of the entire programme.

It should be noted that the problems presented in the previous paragraph are not caused only by policy and managerial factors within the NHES responsible office and the survey programme. As suggested by some informants, the shortfalls were a result of the lack of governance of the health survey system at the national level. Based on such an argument, we can see that the absence of governance is one of the weakest links that hinders the translation of health monitoring surveys, including the NHES, into policy since no institute is authorised to designate the collective priority-setting of survey topics, standardisation of survey methodology, and allocation of scarce resources to a wide range of activities under different health information platforms. Effective coordination among survey projects, if introduced, is likely to be helpful in facilitating communication between policy-makers and experts who conduct policy-relevant analysis of survey data. The introduction of capacity-building programmes on survey data analysis and utilisation, and a mechanism to support the interface between potential data users and offices responsible for the surveys, are examples of actions that can be collectively taken from different health surveys if a corresponding national strategy exists.

## Discussion

Health information is valuable in improving health system performance by guiding health priority-setting, programme designs and implementation [31]. In a national health system context where resources are always constrained, it is the responsibility of governments to ensure that investments in health surveillance offer not only reliable information but also value for money. Compared with household interview surveys, facility-based reporting mechanisms and other information platforms that monitor population health, health examination surveys are relatively expensive as they require substantial budget and time as well as contributions from health professionals, including laboratory workers [32, 33]. This study’s findings on the perspectives of and actions taken by key actors concerning the use of NHES data to inform policy illustrate significant room for improvement.

There are different frameworks to explain the research-policy connection that can be applied to the use of survey data in policy-making [34]. This partly depends on the nature of the evidence generated. Epidemiological data are often used in a conceptual way to gain insight into health problems and determinants, especially the magnitudes, trends, afflicted people and geographical areas [35]. In addition, data from health surveys can be analysed alongside evidence from other sources to demonstrate the performance and benefits of public health interventions [36]. In this study, respective officials in MOPH departments and ThaiHealth as well as NHES office managers were asked to provide concrete examples of NHES-based policy changes. In response, the interviewees did not mention any case of the survey–policy nexus, except for one who commented on the diabetes screening policy. On the other hand, many of the interviewees argued that the NHES data are cited in policy documents such as national strategic plans for disease prevention and annual reports of health agencies. We briefly explored such documents and expanded the search to regional health service plans and National Health Assembly resolutions, and found that most of the citations were located in the introductory sections of the documents and involved discussions on the prevalence of health problems and risk behaviours. Unlike other countries, such as the United States, where health examination survey websites explicitly illustrate the contributions of particular survey programmes to policy agenda-setting and formulation [13], Thailand’s NHES office does not establish such an internet-based communication channel and does not compile any evidence of NHES impact on policy.

Our study indicates that executives and technical officers in MOPH departments and NHES funding agencies are positive about the survey, especially for the technical capacity of the principal researcher, quality of survey data and potential contributions to policy-making. Such a perspective exists despite the limited resources for communication activities. This might be because the NHES is one of the few large survey programmes in the country and has been conducted periodically for over 25 years. Another possible reason is that the interviewees were purposively selected from a list of executives and technical officers who were involved in the fifth cycle of the NHES. This indicates that the NHES, as a long-standing population-based survey with stakeholder participation, does not require substantial investments in wide-coverage dissemination campaigns. However, education programmes to improve understanding about the NHES’s advantages and specific deliverables in comparison to other data sources should be introduced. Additionally, a systematic assessment of NHES uptake, including awareness and acceptance in broader groups of potential policy users, should be raised in future survey programme agenda.

It was also found that although the MOPH and ThaiHealth notably invest in the NHES, the survey findings are rarely used in their planning and programme monitoring and evaluation since alternative surveys are available and provide timely policy-relevant information. Official sources of information for ThaiHealth’s strategic indicators and the collaboration between the MOPH and a university to conduct annual physical activity surveys are illustrative examples. This indicates that NHES funding agencies should review their granting policy and expand it to enhance the return on such a public investment by systematically planning to improve the use of the survey data within and outside their institutes. This suggestion is drawn from the experiences of research funders in six developed countries as they have clear policy and measures to support the translation of research-based evidence into policy [37]. Our study also reflects the need for strengthening the stewardship system for health surveys in Thailand as a means of reducing unnecessary duplication of survey content, creating coordination and integration between survey projects, improving the schedules and timeliness of data released for particular surveys and, ultimately, enhancing efficiency in the use of public resources.

Although only one health officer explicitly mentioned in the interview about the use of NHES data to inform policy at the national level, such arguments should be underpinned on some points. As prevalence of NCDs have been growing in Thailand and elsewhere, early detection and treatment together with enhancing a healthy lifestyle among afflicted cases are important measures. NHES data had depicted silent diabetes attacks in different sub-populations and, therefore, prompted attention of responsible agencies to the diseases and proper interventions. As that example suggests, this health examination survey could play a crucial part in agenda-setting not only for diabetes but also for other NCDs. Moreover, as can be learned from the HSE and NHANES, data from the next cycle of the Thai survey will be helpful in the monitoring and evaluation of the established programmes for NCD management.

As pointed out by public policy scholars, political context and institutional influences, including the prevailing culture for evidence-informed policy, are key factors to enhance the use of evidence in current policy decisions [7, 8]. For Thailand, the literature suggests that, during past decades, evidence from research and other reliable sources have played an important role in policy development, including in the areas of HIV control, universal health coverage, and innovative health promotion funds [4, 38]. From Thai experts’ perspectives, such accomplishments are associated with three factors, namely political engagement, social mobilisation and knowledge management – known as the Triangle that Moves the Mountain model [39]. As such, capacity for evidence generation and translation into policies has been advocated for as crucial elements of rational policy-making and broader health system strengthening [40]. The capacity also involves existing data platforms that can be used in policy research [41]. As suggested in this study, although contextual factors are conducive to an adequate research–policy nexus, Thailand’s room for improvement is substantial, especially in terms of the strategic management of its health information system.

Lessons drawn from Thailand’s NHES can be helpful for policy-makers in other LMICs, as effective governance for evidence generation and utilisation is necessary in all settings regardless of their income level and resources available. As shown in this paper, despite adequate capacity to conduct population health surveys, the lack of governance structure and functions has resulted in crucial weak points, i.e. a fragmented health monitoring system where large and small survey projects are conducted and funded by different institutes without common policy direction and alignment mechanisms for prioritising survey topics, collective planning and capacity-building programmes for survey practitioners and users. In the context of introducing Sustainable Development Goals, both facility-based reporting mechanisms and health surveys should play a role in monitoring and measuring the progress and achievements of the Sustainable Development Goals’ health components at the national level [42, 43]. This means that significant amounts of resources will be mobilised to M&E, including health surveillance, without governance and coordination in the government and academic sector – a wasteful use of resources can be anticipated.

An important limitation of this study is that it largely relies on interview data based on key informants’ perspectives and experiences. Furthermore, the study’s conceptual framework was not designed to capture formal and informal interactions between NHES funding agencies, survey practitioners and policy-makers, as potential users of the survey data. Additionally, the framework does not address different ways of data utilisation, as evidence obtained from the survey may be used straightforwardly to guide programme development while, in some instances, the survey can raise awareness on certain health problems among decision-makers and key stakeholders. Although the latter case may lead to policy change in later steps, it is difficult to determine if such a development is attributed to the NHES or other factors. As such, this framework seems to be inadequate to explain policy shifts if the connection between the two elements is non-linear, such as those that fall into the social interaction model, enlightenment model or tactical model as reviewed by Hanney et al. [6]. For future research to determine the implications of NHES data on specific policy decisions, public policy theories and models such as policy transfer [44], policy networks and communities [45], and the policy advocacy coalition framework [46] provide a more relevant basis to the study’s conceptual framework and data gathering tools.

## Conclusions

Despite an adequate capacity to conduct population health surveys, the lack of governance structure and function has resulted in a fragmented health monitoring system. Resource constraints for NHES dissemination campaigns as well as the lack of a proper mechanism to identify and respond to policy questions were factors affecting limited use of the survey evidence in policy-making. These problems are caused not only by policy and managerial factors within the NHES responsible office and the survey programme. The shortfalls are a result of the lack of governance of the health survey system at the national level. The absence of governance is one of the weakest links that hinders the translation of health monitoring surveys, including the NHES, into policy since no institute is authorised to designate the collective priority-setting of survey topics, standardisation of survey methodology and allocation of scarce resources to a wide range of activities under different health information platforms. Lessons drawn from Thailand’s NHES can be helpful for policy-makers in other LMICs, as effective governance for evidence generation and utilisation is necessary in all contexts regardless of income level and available resources.

## Data Availability

The datasets used and analysed during the current study are available from the corresponding author on reasonable request.

## Abbreviations

HITAP: Health Intervention and Technology Assessment Program
HSE: Health Survey for England
HSRI: Health Systems Research Institute
LMICs: low- and middle-income countries
M&E: monitoring and evaluation
MOPH: Ministry of Public Health
NCDs: non-communicable diseases
NHANES: National Health and Nutrition Examination Survey
NHES: National Health Examination Survey
ThaiHealth: Thai Health Promotion Foundation
